# Indoleamine 2,3-dioxygenase 2 immunohistochemical expression in medullary thyroid carcinoma: implications in prognosis and immunomodulatory effects

**DOI:** 10.1186/s12885-022-10173-7

**Published:** 2022-11-01

**Authors:** Pengfei Gu, Bin Ling, Weike Ma, Jinming Zhang, Wei Zhang, Yu Zeng, Yu Liu, Jiadong Chi, Xianhui Ruan, Xiangqian Zheng, Songfeng Wei, Ming Gao

**Affiliations:** 1 Department of Thyroid and Neck Cancer, Key Laboratory of Cancer Prevention and Therapy, Tianjin Medical University Cancer Institute and Hospital, National Clinical Research Center for Cancer, Tianjin’s Clinical Research Center for Cancer, 300060 Tianjin, China; 2 Cancer Precision Medicine Center, Tianjin Cancer Hospital Airport Hospital, Tianjin, China; 3 Center For Precision Cancer Medicine & Translational Research, Tianjin Medical University Cancer Institute & Hospital, Tianjin, China; 4 Department of Thyroid and Breast Surgery, Tianjin Union Medical Center, 300121 Tianjin, China; 5 Tianjin Key Laboratory of General Surgery in construction, Tianjin Union Medical Center, 300121 Tianjin, China

## Abstract

**Background:**

The linkage between IDO2 expression and cancer progression is still unclear, particularly in medullary thyroid carcinoma (MTC). Our purpose is to unveil the potential correlations between IDO2 status, clinical-pathological parameters, patients’ prognosis, and the possible immunomodulatory functions in MTC.

**Methods:**

Immunohistochemical expression levels of IDO2 were evaluated in the resected MTC surgical specimens and corresponding lymph nodes. CD4 + T cell infiltration was also evaluated by immunohistochemical analysis in the MTC tissues. The association of the IDO2 expression level with clinicopathologic characteristics, overall survival (OS)/recurrence-free survival (RFS), and CD4 + T cell infiltration were retrospectively investigated.

**Results:**

High expression of IDO2 is closely associated with more aggressive clinicopathological features, such as multifocality, ETE, a higher pT stage and especially a higher pN stage. Moreover, a significant difference in RFS was observed between the IDO2-high and IDO2-low groups. IDO2 expression of lymph node tissues was significantly related to the metastasis status. Furthermore, we found that IDO2 expression is negatively correlated with CD4 + T cell infiltrations in MTC tissues.

**Conclusion:**

The expression level of IDO2 is associated with aggressive characteristics and is predictive of poor prognosis in patients with MTC. Also, an interesting observation is that IDO2 involvement in MTC showed a moderate sexual dimorphism, of which female patients tend to be more affected by IDO2 status. Moreover, our results showed the potential immunomodulatory functions of IDO2. The close relationship between IDO2 and CD4 + T cell infiltration in the MTC microenvironment, together with its potential prognostic implications, makes it possible for IDO2 to serve as an alternative drug target in cancer immunotherapy and as a new prognostic tool.

## Introduction

Medullary thyroid carcinoma (MTC) is a rare malignancy that originates from parafollicular cells (C cells) of the thyroid and accounts for 2–4% of all thyroid malignancies [1]. Despite its low prevalence, MTC demonstrates a more aggressive clinical course and has a higher prevalence of advanced disease at diagnosis than differentiated thyroid cancer [2, 3]. The 10-year survival rate decreases from 96% in patients with tumors confined to the thyroid to 75%~40% with regional and distant metastasis, respectively [4, 5]. Moreover, no curative therapies are effective for patients with locally/locoregionally advanced inoperable disease and/or distant metastases [6]. Effective additional or alternative treatment is still urgently needed.

A significant amount of evidence indicates that tryptophan (Trp) metabolism is of paramount importance in cancer progression and for the increasing malignancy of cancer cells [7–9]. In mammals, there are three dioxygenases that catalyze Trp into kynurenine, including indoleamine 2,3-dioxygenase 1 (IDO1), IDO2, and tryptophan 2,3-dioxygenase (TDO; [10, 11]. It has been shown that IDO1, the most important enzyme that catalyzes the initial, rate-limiting step in the degradation of Trp along the kynurenine pathway, is highly expressed in many types of human cancers, including endometrial [12], colon [13], and epithelial ovarian carcinomas [14], and IDO1 is generally associated with poor prognosis [15]. Moreover, several studies have shown that IDO1 plays a significant role in facilitating naive T lymphocytes into T regulatory cells [16, 17], and IDO1 expression can foster an immunotolerant environment [18–20]. For this reason, IDO1 catalytic inhibitors have been used as experimental drugs in cancer immunotherapy [21]. Consistent with the role of IDO1 in mediating tolerance to tumors, preclinical studies have shown that IDO1 inhibitors have a promising prospect in targeting several cancers [22–24]. However, a recent phase III clinical trial on one of these inhibitors, epacadostat, failed to show the efficacy observed in the previous phase II trial [25]. Considering the complex functional dynamics, it may be difficult for IDO1 to serve as an effective drug target.

IDO2, a paralog of IDO1, was recently discovered through searching a high-throughput sequencing library by Ball et al. in 2007 [26]. IDO1 and IDO2 are closely linked on chromosome 8 in humans, probably originating from an ancient gene duplication that occurred prior to the evolution of vertebrates [11, 27]. Although characterized by a high level of sequence identity, IDO2 affinity for the Trp substrate and catalytic efficacy in producing kynurenine are very low or almost negligible [28]. However, IDO2 does not simply serve a redundant function to IDO1 [29]. It was found that the expression of IDO2 is dysregulated in a variety of cancers. In most cancers, the expression of IDO2 is upregulated, including non-small-cell lung cancer [30], pancreatic cancer [31], colon cancer, gastric cancer, and renal tumors [32]. Moreover, similar to IDO1, IDO2 was also reported to inhibit the proliferation of CD4 + and CD8 + T cells [33, 34]. Furthermore, IDO2 is involved in the generation of T regulatory cells induced by DCs [35, 36]. This indicates that IDO2 may play a role on tumor immune resistance and may thus be regarded as a potential target for cancer immunotherapy.

The linkage between IDO2 expression and cancer progression is still unclear, particularly in MTC. In the current study, we evaluate the level of IDO2 through its immunohistochemical expression by using a large cohort of patients with MTC to unveil its correlation with clinicopathologic factors, OS/recurrence-free survival (RFS), and biochemical recurrence. Furthermore, we also evaluated the correlation between IDO2 and the CD4 + T expression of this relatively rare malignancy to outline IDO2 as both a potential new biomarker for better patient risk stratification and as a possible target for the immune therapy of MTC.

## Materials and methods

### Patients and samples

Patients were recruited from the computer archive of the Institute of Pathology and Histology, Tianjin Medical University Cancer Institute and Hospital, Tianjin, China, involving all the first diagnosed MTC cases that underwent a surgical resection in the period from January 2011 to December 2019. The inclusion criteria for this study included the following: (i) patients with histologically proven MTC; (ii) patients who underwent initial surgery with a lymphadenectomy in our institution; (iii) patients with pathologically negative resection margins (R0 resection); and (iv) patients with available formalin-fixed, paraffin‐embedded tumor tissues with the corresponding metastatic or nonmetastatic lymph node tissues. The exclusion criteria were as follows: (i) patients with a history of thyroidectomy or other malignancy; (ii) patients who received chemotherapy or radiotherapy before surgery; and (iii) patients with no available clinicopathologic data and medical history. Before the study began, all available hematoxylin‐eosin (HE)-stained and immunohistochemical slides were reexamined by two experienced pathologists to confirm the diagnosis of MTC according to the current World Health Organization criteria.

Ultimately, 187 MTC patients were included in the study. This study was approved by the Institutional Review Board of Tianjin Medical University Cancer Institute and Hospital; informed consent was provided from each patient. The demographics, tumor characteristics, treatment details, pathologic findings, and imaging information of the patients were obtained.

### Patients management

Patients underwent total thyroidectomy or hemithyroidectomy with systematic lymphadenectomy. Central compartment (level VI) neck dissection was routinely performed for thyroid malignancies in our institution. Modified lateral neck and/or upper mediastinal lymphadenectomy was performed if there were biopsy-proven or preoperatively suspicious lateral neck and/or level VII metastases. After undergoing curative surgery, all 187 patients were followed up every 3 or 6 months for 2 years, then every 6 months for the next 3 years, and annually thereafter until death. The follow-up period for each patient was defined as the length of time from the initial surgery until the last known contact, either by telephone contact or by review of inpatient or outpatient medical records at our institution. The follow-ups of all patients included in this study were completed in December 2020.

All patients received TSH-suppressive hormonal therapy following surgery. Recurrence was defined as the appearance of disease, with new biopsy-proven/secondary surgery–confirmed local disease or distant disease revealed by US and/or imaging scans in any patient who had been free of disease (no palpable disease). Local/distant RFS was defined as the time between the date of initial surgery and the first event of recurrence or death. A biochemical cure was defined as a postoperative basal Ctn level within the reference range and with no signs of recurrence/distant metastasis found by US and/or imaging scans at the same time.

### Clinicopathologic variables assessed

Clinicopathologic variables, such as gender, age at diagnosis, maximal tumor size, multifocal lesions (defined as two or more cancer sites within the thyroid; multifocal lesions could be either unilateral or bilateral), extrathyroidal extension (ETE), coexistent Hashimoto’s thyroiditis (HT) and lymph node metastasis were collected for analysis. The eighth edition of the American Joint Committee on Cancer (AJCC) Cancer Staging Manual was utilized for tumor staging.

### Immunohistochemistry and evaluation

Tissue sections were obtained from 187 formalin-fixed, paraffin-embedded MTC tumor specimens and corresponding lymph nodes. IDO2 and CD4 expression was evaluated by immunohistochemistry (IHC). All immunohistochemistry assays included negative controls with the primary antibodies omitted and positive controls including tonsils and lymph nodes. After antigen retrieval by microwaving, IHC staining was performed on 4 μm-thick, paraffin-embedded serial sections of tissue samples using human anti-IDO2 (TA806648, OriGene, working dilution 1:200) and human anti-CD4 (ab183685, Abcam, working dilution 1:1,000) antibodies according to the manufacturer’s instructions. Each primary antibody-probed section was incubated with the corresponding secondary antibody, followed by a horse-radish peroxidase-labeled streptavidin reaction manually. Immune complexes were detected using 3,3′-diaminobenzidine (DAB) tetrahydrochloride chromogen mixed with DAB substrate, and the sections were counterstained with hematoxylin and coverslipped.

**Fig. 1:**
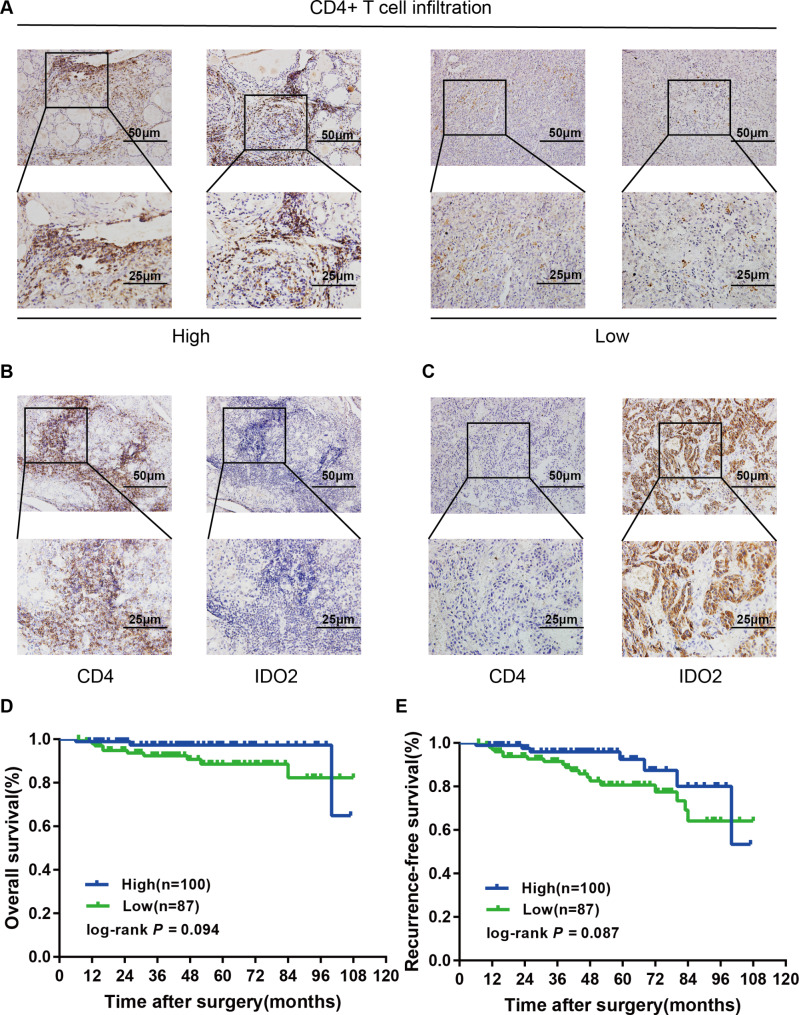
Correlation between IDO2 expression in MTC tissues and clinicopathologic features. (A) Representative immunohistochemistry images showing low and high expression of IDO2 in MTC tissues (top, 200x; below, 400x). (B) Box plot showing the association of IDO2 expression with clinicopathologic features (age at surgery, gender, hereditary status, multifocality, bilateral lesions, tumor size, pT stage, pN stage, 8th TNM stage, coexisting Hashimoto’s thyroiditis)

The IHC-stained tissue sections were scored separately by two pathologists blinded to the clinical parameters. In cases of disagreement, the result was reached by consensus. The Positive staining for IDO2 showed brownish yellow particles in the cytoplasm and cytomembrane of the cells. As for the evaluation of IDO2 expression, the staining intensity was scored as 0 (none), 1 (weak), 2 (medium), or 3 (strong). The extent of the staining was scored as 0, < 5%; 1, 5–25%; 2, 26–50%; 3, 51–75%; and 4, > 75% according to the percentages of the positive staining areas in relation to the whole carcinoma area. Scores for staining intensity and percentage of positivity cells were then multiplied to generate the immunoreactivity score for each case. As for the determination of the cutoff value, the receiver operating characteristic (ROC) curve with a Youden index was used. After statistical analysis, the cutoff is determined to be 4. Therefore, tissues having a final staining score of < 4 or 4–12 were considered to be low or high staining, respectively (Fig. 1). IHC staining protocol and evaluation of the corresponding lymph nodes was identical to the MTC tissues.

As for the evaluation of CD4 expression, the staining results were classified by the number of immune-reactive cells (highlighted by membrane staining). Five images from representative lymphocyte-enriched regions at 40 × magnification were collected, and the numbers of positively stained cells were counted by two pathologists separately. The cut-off value was also determined using the ROC curve with a Youden index. Patients with cell numbers greater than the cut-off value were defined as “high” and those with smaller cell numbers were defined as “low.”

### Statistical analysis

All statistical analyses were performed using IBM SPSS Statistics (Version 26.0; IBM Corp, New York, USA). Chi-square/Fisher Exact test and t-test/Manne-Whitney U test were used for categorical and continuous variables, respectively. Patients were divided into a young and an elderly group, according to the cutoff age (52 years, corresponding to patients’ median age) for analysis. Categorical variables were compared between the groups (low IDO2 or high IDO2) using a chi-squared test or Fisher’s exact test as appropriate. The probabilities of OS and RFS were calculated by the Kaplan–Meier method, and the differences in OS/RFS probabilities between groups were examined by log-rank tests. Multivariate Cox proportional-hazards regression was used to evaluate the independent prognostic risk factors of OS and to calculate hazard ratios (HRs) with 95% confidence intervals (CIs). Logistic regression analysis was used to evaluate the risk factors of lymph node metastasis. The relationship between two variables were evaluated using the Pearson correlation test or Spearman correlation test. In all analyses, two-tailed *P* < 0.05 was considered statistically significant.

## Results

### Characteristics of patients

For the overall cohorts, the median age was 52 years (range 14–74), with a median follow-up period of 46 months (range 12–107 months). Eighty-five (45.5%) patients were males. Thirty-three (17.6%) patients presented in a hereditary pattern, and the reminder presented in a sporadic form. During the follow-up period, 98 (52.4%) patients achieved a biochemical cure, whereas 22 (11.8%) patients relapsed after surgery. Thirteen (6.9%) patients died from MTC.

According to the final pathology, 100 patients (53.5%) presented lymph node metastasis, among whom 74(39.6%) metastasized to lateral neck and/or upper mediastinum. Seventy-six (40.6%) patients were diagnosed with tumor extending beyond the thyroid into the surrounding tissues. Bilateral disease was identified in 20 (10.7%) patients, whereas multifocal disease was noted in 55 (29.4%) patients. Regarding the 8th AJCC TNM staging classification, 46 cases (24.6%) belonged to stage I, 31 cases (16.6%) belonged to stage II, 22 cases (11.8%) belonged to stage III, and 88 cases (47.1%) patients were in stage IV. The characteristics of these patients are shown in Table 1.

**Table 1:** Association of IDO2 expression with clinicopathological characteristics of MTC

Characteristics	***N*** (%)			***P*** value
	**Total**	**Low IDO2 scores**	**High IDO2 scores**	
**Gender**				**0.017** ^*^
Male	85(45.5)	42(49.4)	43(50.6)	
Female	102(54.5)	68(66.7)	34(33.3)	
**Age at surgery(year)**				0.472
≤ 52	101(54.0)	57(56.4)	44(43.6)	
>52	86(46.0)	53(61.6)	33(38.4)	
**Heredity**				0.582
Hereditary	33(17.6)	18(54.5)	15(45.5)	
sporadic	154(82.4)	92(59.7)	62(40.3)	
**Multifocal**				**0.038** ^*^
Yes	55(29.4)	26(47.3)	29(52.7)	
No	132(70.6)	84(63.6)	48(36.4)	
**ETE**				**0.020** ^*^
Yes	76(40.6)	37(48.7)	39(51.3)	
No	111(59.4)	73(65.8)	38(34.2)	
**Bilateral lesions**				0.184
Yes	20(10.7)	9(45%)	11(55%)	
No	167(89.3)	101(60.5)	66(39.5)	
**Tumor size(cm)**				0.716
≤ 2	120(64.2)	73(60.8)	47(39.2)	
2–4	57(30.5)	31(54.4)	26(45.6)	
>4	10(5.3)	6(60.0)	4(40.0)	
**pT stage**				**0.004** ^*^
T1/T2	108(57.7)	73(67.6)	35(32.4)	
T3/T4	79(42.3)	37(46.8)	42(53.2)	
**pN stage**				**<0.001** ^*^
N0	87(46.5)	73(83.9)	14(16.1)	
N1a	26(13.9)	10(38.5)	16(61.5)	
N1b	74(39.6)	27(36.5)	47(63.5)	
**8th TNM stage**				**<0.001** ^*^
I/II	77(41.1)	63(81.8)	14(18.2)	
III/IV	110(58.9)	47(42.7)	63(57.3)	
**Coexist HT**				0.403
Yes	24(12.8)	16(66.7)	8(33.3)	
No	163(87.2)	94(57.7)	69(42.3)	
**Pathology**				0.319
Only MTC	105(56.1)	58(55.2)	47(44.8)	
With adenomatous goiter	9(4.8)	6(66.7)	3(33.3)	
With nodular goiter	51(27.3)	35(68.6)	16(31.4)	
With PTC	22(11.8)	11(50.0)	11(50.0)	
**Recurrence**				**0.023** ^*^
Yes	22(11.8)	8(36.4)	14(63.6)	
No	165(88.2)	102(61.8)	63(38.2)	
**Biochemical cure**				**0.002** ^*^
Yes	98(52.4)	68(69.4)	30(30.6)	
No	89(47.6)	42(47.2)	47(52.8)	

^*^P<0.05; MTC, medullary thyroid carcinoma; IDO2, indoleamine 2,3-dioxygenase 2; ETE, extrathyroid extension; HT, Hashimoto’s thyroiditis

### Correlation between IDO2 expression in cancer tissues and clinicopathologic features

The expression levels of IDO2 in MTC tissues were analyzed by IHC staining. IDO2 expression was high in 77/187 (41.2%) MTC samples and low in the remaining 110 (58.8%) samples. Representative images are shown in Fig. 1 A. The association of IDO2 expression with clinicopathologic features and outcomes was determined in MTC tissues (Table 1). IDO2 expression was positively associated with female gender (*P* = 0.017), multifocality (*P* = 0.038), ETE (*P* = 0.020), pT stage (*P* = 0.019), pN stage (*P* < 0.001), and 8th AJCC pTNM stage (*P* < 0.001), but it was not correlated with age, bilateral lesions, maximum tumor size, and coexisting HT (*P* > 0.05; Fig. 1B). In particular, high expression of IDO2 was significantly associated with disease recurrence and biochemical cure (*P* = 0.023 and 0.002, respectively).

### Correlation between IDO2 expression in cancer tissues and patients’ survival

**Fig. 2:**
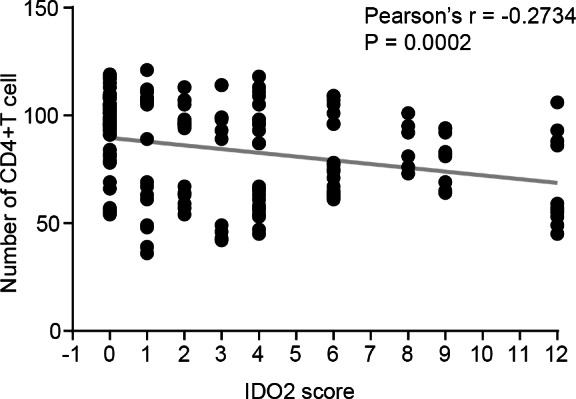
Kaplan–Meier survival plots presenting overall survival of (A) the whole cohort, (C) male patients, and (E) female patients and recurrence-free survival of (B) the whole cohort, (D) male patients, and (F) female patients

The Kaplan–Meier survival analysis demonstrated that the 5-year survival rate of the overall cohort was 92.4%, whereas the 5-year survival rate of the IDO2-high and IDO2-low groups were 88.9% and 95.0%, respectively. In the log-rank test, no significant difference was observed between the two groups (*P* = 0.274; Fig. 2 A). Furthermore, the 5-year RFS of the overall cohort was 85.2%, and the 5-year RFS of the IDO2-high and IDO2-low groups were 81.4% and 89.5%, respectively. In the log-rank test, a significant difference in RFS was observed between the high and low groups (*P* = 0.022; Fig. 2B).

Univariate analysis revealed that the following six clinicopathological characteristics were significantly associated with RFS in the MTC patients: multifocality, extrathyroidal extension, pT stage, pN stage, pTNM stage, and IDO2 expression (Table 2). These characteristics were then included in a multivariate Cox proportional hazard model (forward stepwise procedure) to adjust for the effects of covariates. Multivariate analysis indicated that only the pN stage (HR, 1.371; *P* = 0.007) was an independent predictor of RFS in MTC patients (Table 2).

**Table 2:** Univariable and multivariate Cox analysis for RFS in patients with MTC

Characteristics	Univariable analysis			Multivariable analysis		
	**HR**	**95% CI**	***P***	**HR**	**95% CI**	***P***
Gender	0.833	0.386–1.798	0.641			
Age at diagnose (52)	0.581	0.259–1.304	0.188			
Multifocal	2.340	1.078–5.079	**0.032** ^*^	1.366	0.601–3.107	0.456
ETE	2.961	1.315–6.666	**0.009** ^*^	2.226	0.334–14.846	0.408
Bilateral lesions	1.614	0.555–4.691	0.380			
Tumor size	1.680	0.933–3.026	0.084			
pT stage	1.545	1.113–2.143	**0.009** ^*^	0.880	0.379–2.044	0.766
pN stage	2.235	1.374–3.636	**0.001** ^*^	1.371	0.540–3.482	**0.007** ^*^
AJCC 8th TNM stage	1.975	1.265–3.084	**0.003** ^*^	1.186	0.494–2.847	0.702
Coexist HT	1.745	0.412–7.389	0.450			
Pathology	0.821	0.541–1.246	0.353			
IDO2 expression	2.530	1.147–5.577	**0.021** ^*^	1.562	0.639–3.823	0.328

^*^P<0.05; RFS, recurrence-free survival; MTC, medullary thyroid carcinoma; HR, hazard ratio; CI, confidence interval; IDO2, indoleamine 2,3-dioxygenase 2; ETE, extrathyroid extension; HT, Hashimoto’s thyroiditis

Interestingly, we found that IDO2 involvement in MTC showed a moderate sexual dimorphism. IDO2 staining status was evenly distributed in male MTC patients, whereas the female counterparts were more likely to be manifested as a IDO2-low status (Table 1). The characteristics stratified by gender are summarized in Table 3. The pN stage was higher in male patients (*P* < 0.001), but there was no difference in recurrence or biochemical cure rates (both *P* = 0.649). Moreover, in the log-rank test, no significant difference in RFS was observed in the male MTC patients according to the IDO2 expression (*P* = 0.397; Fig. 2D), whereas the IDO2-low group showed a better prognosis than the IDO2-high group in female MTC patients (*P* = 0.016; Fig. 2 F). However, no significant difference in OS was observed between the IDO2-low group and IDO2-high group when stratified by gender (*P* = 0.752 and 0.356, respectively; Fig. 2 C and 2E). Therefore, these data would suggest that more attention should be paid to the expression status of IDO2 in female patients with MTC.

**Table 3:** Association of gender with clinicopathological characteristics of MTC

Characteristics	***N*** (%)			***P*** value
	**Total**	**Male**	**Female**	
**Age at surgery(year)**				0.391
≤ 52	101(54.0)	43(42.6)	58(57.4)	
>52	86(46.0)	42(48.8)	44(51.2)	
**Heredity**				0.122
Hereditary	33(17.6)	18(54.5)	15(45.5)	
sporadic	154(82.4)	92(59.7)	62(40.3)	
**Multifocal**				0.334
Yes	55(29.4)	28(50.9)	27(49.1)	
No	132(70.6)	57(43.2)	75(56.8)	
**ETE**				0.302
Yes	76(40.6)	38(50.0)	38(50.0)	
No	111(59.4)	47(42.3)	64(57.7)	
**Bilateral lesions**				0.666
Yes	20(10.7)	10(50.0%)	10(50.0%)	
No	167(89.3)	75(44.9)	92(55.1)	
**Tumor size(cm)**				0.572
≤ 2	120(64.2)	55(45.8)	65(54.2)	
2–4	57(30.5)	24(42.1)	33(57.9)	
>4	10(5.3)	6(60.0)	4(40.0)	
**pT stage**				0.358
T1/T2	108(57.7)	46(42.6)	62(57.4)	
T3/T4	79(42.3)	39(49.4)	40(50.6)	
**pN stage**				**<0.001** ^*^
N0	87(46.5)	28(32.2)	59(67.8)	
N1a	26(13.9)	12(46.2)	14(53.8)	
N1b	74(39.6)	45(60.8)	29(39.2)	
**8th TNM stage**				**<0.001** ^*^
I/II	77(41.1)	21(27.3)	56(72.7)	
III/IV	110(58.9)	64(58.2)	46(41.8)	
**Coexist HT**				0.086
Yes	24(12.8)	7(29.2)	17(70.8)	
No	163(87.2)	78(47.9)	85(52.1)	
**Pathology**				0.290
Only MTC	105(56.1)	53(50.5)	52(49.5)	
With adenomatous goiter	9(4.8)	5(55.6)	4(44.4)	
With nodular goiter	51(27.3)	18(35.3)	33(64.7)	
With PTC	22(11.8)	9(40.9)	13(59.1)	
**Recurrence**				0.649
Yes	22(11.8)	11(50.0)	11(50.0)	
No	165(88.2)	74(44.8)	91(55.2)	
**Biochemical cure**				0.649
Yes	98(52.4)	43(43.9)	55(56.1)	
No	89(47.6)	42(47.2)	47(52.8)	
**IDO2 expression**				**0.017** ^*^
High	77(41.1)	43(55.8)	34(44.2)	
Low	110(58.9)	42(38.2)	68(61.8)	

^*^P<0.05; MTC, medullary thyroid carcinoma; IDO2, indoleamine 2,3-dioxygenase 2; ETE, extrathyroid extension; HT, Hashimoto’s thyroiditis

### IDO2 expression in corresponding lymph nodes

**Fig. 3:**
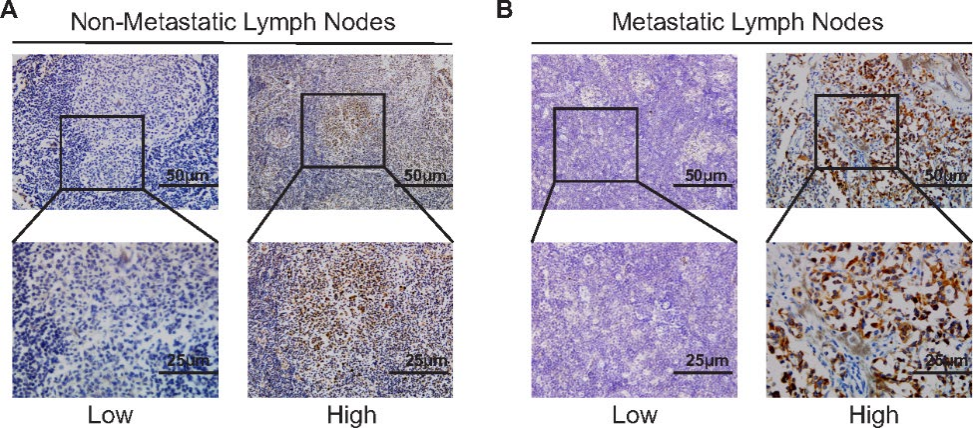
Representative immunohistochemistry images showing low and high expression of IDO2 in (A) nonmetastatic lymph nodes and (B) metastatic lymph nodes (Top, 200x; below, 400x)

MTC patients with lymph node metastasis tend to have higher levels of IDO2 expression (*P* < 0.001, Table 1). Moreover, logistic regression analysis revealed that the following clinicopathological characteristics were significantly related with lymph node metastasis in MTC patients: gender, heredity, multifocality, ETE, pT stage, and IDO2 expression level (odds ratio, 8.878; 95% CI, 4.403 to 17.902; *P* = 0.001; Table 4). To further unveil the relationship between IDO2 expression and lymph node metastasis in MTC patients, IHC staining of IDO2 was performed on the corresponding lymph node tissues. Well-preserved corresponding metastatic lymph nodes and nonmetastatic lymph nodes represented the MTC patients with or without lymph node metastasis, respectively. Representative images are shown in Fig. 3 A and 3B. Our results indicated that the IDO2 expression of lymph node tissues was significantly related to the metastasis status. Correlation analysis revealed a statistically significant correlation between the above two parameters (Spearman’s r = 0.227, *P* = 0.002; Table 5). Interestingly, our results also indicated that there was a significant correlation between the expression level of IDO2 in MTC tissues and the corresponding lymph node tissues (Spearman’s r = 0.268, *P* = 0.022; Table 5). Collectively, these data suggests that IDO2 may play an important role in promoting lymph node metastasis in MTC patients, of which the mechanism needs to be further discussed.

**Table 4:** Risk of developing lymph node metastasis according to clinicopathologic factors and IDO2 expression

Characteristics		***N*** (%)		OR (95% CI)	***P***-value
	**Total**	**Without lymph node metastasis**	**With lymph node metastasis**		
**Gender**				0.358(0.197–0.652)	**0.001** ^*^
Male	85(45.5)	28(32.9)	57(67.1)		
Female	102(54.4)	59(57.8)	43(42.2)		
**Age at diagnose(year)**				0.918(0.516–1.634)	0.771
≤ 52	101(54.0)	46(45.5)	55(54.5)		
>52	86(46.0)	41(47.7)	45(52.3)		
**Heredity**				2.312(1.089–4.906)	**0.029** ^*^
Hereditary	39(20.8)	12(30.8)	27(69.2)		
sporadic	148(79.2)	75(50.7)	73(49.3)		
**Multifocal**				2.837(1.444–5.572)	**0.002** ^*^
Yes	55(29.4)	16(29.1)	39(70.9)		
No	132(70.6)	71(53.8)	61(46.2)		
**ETE**				2.844(1.542–5.246)	**0.001** ^*^
Yes	76(40.6)	24(31.6)	52(68.4)		
No	111(59.4)	63(56.8)	48(43.2)		
**Bilateral lesions**				1.347(0.524–3.464)	0.537
Yes	20(10.7)	8(40.0)	12(60.0)		
No	167(89.3)	79(47.3)	88(52.7)		
**Tumor size(cm)**				1.052(0.646–1.712)	0.838
≤ 2	120(64.2)	56(46.7)	64(53.3)		
2–4	57(30.5)	27(47.4)	30(52.6)		
>4	10(5.3)	4(40.0)	6(60.0)		
**pT stage**				2.911(1.584–5.350)	**0.001** ^*^
T1/T2	108(57.7)	62(57.4)	46(42.6)		
T3/T4	79(42.3)	25(31.6)	54(68.4)		
**Coexist HT**				0.654(0.271–1.579)	0.345
Yes	24(12.8)	9(37.5)	15(62.5)		
No	163(87.2)	78(47.9)	85(52.1)		
**IDO2 expression**				8.878(4.403–17.902)	**0.001** ^*^
High	77(41.1)	14(18.2)	63(81.8)		
Low	110(58.9)	73(66.4)	37(33.6)		

^*^P<0.05; OR, odds ratio; CI, confidence interval; IDO2, indoleamine 2,3-dioxygenase 2; ETE, extrathyroid extension; HT, Hashimoto’s thyroiditis

**Table 5:** The relationship between the expression of IDO2 in MTC tissues/corresponding lymph node tissues, lymph node metastasis, and CD4 + T cell infiltrations

	Total	***N*** (%)		Spearman’s ***r***	***P***
		LN high expression	LN low expression	0.227	**0.002** ^*^
With LN metastasis	100(53.5)	46(46.0)	54(54.0)		
Without LN metastasis	87(46.5)	21(24.1)	66(75.9)		
		LN high expression	LN low expression	0.268	**0.022** ^*^
Tissue high expression	77(41.2)	35(45.5)	42(54.5)		
Tissue low expression	110(58.8)	32(29.1)	78(70.9)		
		Tissue high expression	Tissue low expression	-0.243	0.001*
CD4 low	87(6.5)	47(54.0)	40(46.0)		
CD4 high	100(53.5)	30(30.0)	70(70.0)		

^*^P<0.05; MTC, medullary thyroid carcinoma; IDO2, indoleamine 2,3-dioxygenase 2

### IDO2 expression is negatively correlated with CD4 expression in MTC tissues

**Fig. 4:**
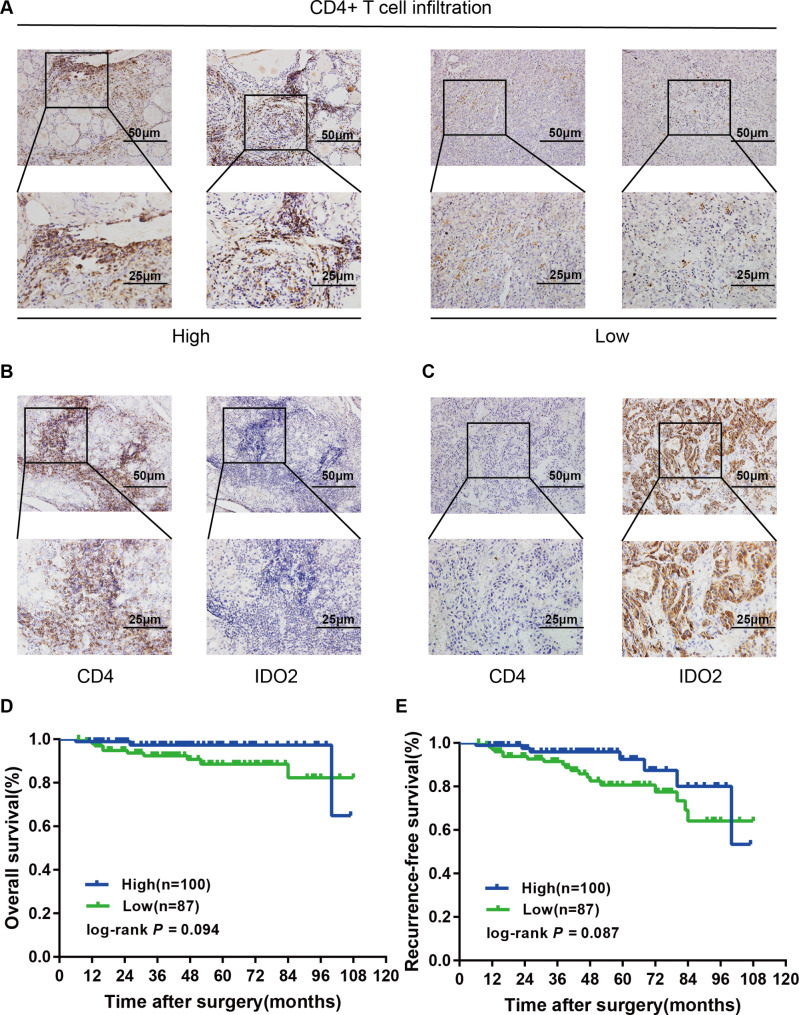
Association between CD4 + T cell infiltration and prognosis of MTC. Representative immunohistochemistry images showing (A) low and high expression of CD4 + T cell in MTC tissues. (B, C) obviously opposite expression of IDO2 and CD4 in a same MTC patient (Left, CD4-low and IDO2-high; right, CD4-high and IDO2-low; top, 200x; below, 400x). Kaplan–Meier survival plots presenting overall survival (D) and recurrence-free survival (E) of the whole cohort

A number of studies have focused on the effects of IDO2 on tumor immune resistance [31, 33, 34, 36, 37]. Similar to IDO1, IDO2 was also reported to inhibit the proliferation of CD4 + and CD8 + T cells. Concerning with this, the infiltration of CD4 + T cells in MTC tissues was evaluated by IHC staining. Representative images are shown in Fig. 4 A. The association of CD4 expression with clinicopathologic features in MTC tissues was summarized in Table 6. CD4 + T cell infiltration was positively associated with the hereditary (*P* = 0.027) and IDO2 expression level (*P* = 0.001). Unlike the role of IDO2, we found no significant correlation between the CD4 + T cell infiltration and other clinicopathological features of patients with MTC. Furthermore, the expression of CD4 had no significant effect on recurrence and biochemical cure in the MTC patients. Similarly, the Kaplan–Meier survival analysis and the log-rank test showed no significant difference in OS and RFS between the CD4-high and -low groups (*P* = 0.094 and 0.087, respectively; Fig. 4D and E).

**Table 6:** Association between CD4 + T cell infiltration and clinicopathological features of MTC

Characteristics	***N*** (%)			***P*** value
	**Total**	**CD4 Low**	**CD4 High**	
**Gender**				0.649
Male	85(45.5)	38(44.7)	47(55.3)	
Female	102(54.5)	49(48.0)	53(52.0)	
**Age at diagnose(year)**				0.766
≤ 52	101(54.0)	48(47.5)	53(52.5)	
>52	86(46.0)	39(45.3)	47(54.7)	
**Heredity**				**0.027** ^*^
Hereditary	39(20.8)	12(30.8)	27(69.2)	
sporadic	148(79.2)	75(50.7)	73(49.3)	
**Multifocal**				0.405
Yes	55(29.4)	23(41.8)	32(58.2)	
No	132(70.6)	64(48.5)	68(51.5)	
**ETE**				0.481
Yes	76(40.6)	33(43.4)	43(56.6)	
No	111(59.4)	54(48.6)	57(51.4)	
**Bilateral lesions**				0.196
Yes	20(10.7)	7(35.0)	13(65.0)	
No	167(89.3)	84(50.3)	8349.7)	
**Tumor size(cm)**				0.063
≤ 2	120(64.2)	52(43.3)	68(56.7)	
2–4	57(30.5)	33(57.9)	24(42.1)	
>4	10(5.3)	2(20.0)	8(80.0)	
**pT stage**				0.414
T1/T2	108(57.8)	53(49.1)	55(50.9)	
T3/T4	79(42.2)	34(43.0)	45(57.0)	
**pN stage**				0.098
N0	87(46.5)	36(41.4)	51(58.6)	
N1a	26(13.9)	17(65.4)	9(34.6)	
N1b	74(39.6)	34(45.9)	40(54.1)	
**8th TNM stage**				0.587
I/II	77(41.2)	34(44.2)	43(55.8)	
III/IV	110(58.8)	53(48.2)	57(51.8)	
**Coexist HT**				0.609
Yes	24(12.8)	10(41.7)	14(58.3)	
No	163(87.2)	77(47.2)	86(52.8)	
**Pathology**				0.369
Only MTC	105(56.1)	3(41.0)	62(59.0)	
With adenomatous goiter	9(4.8)	5(55.6)	4(44.4)	
With nodular goiter	51(27.3)	28(54.9)	23(45.1)	
With PTC	22(11.8)	11(50.0)	11(50.0)	
**Recurrence**				0.141
Yes	22(11.8)	7(31.8)	15(68.2)	
No	165(88.2)	80(48.5)	85(51.5)	
**Biochemical cure**				0.112
Yes	98(52.4)	51(52.0)	47(48.0)	
No	89(47.6)	36(40.4)	53(59.6)	
**IDO2 expression**				0.001*
Low	110(58.8)	40(36.4)	70(63.6)	
High	77(41.2)	47(61.0)	30(39.0)	

^*^P<0.05; MTC, medullary thyroid carcinoma; IDO2, indoleamine 2,3-dioxygenase 2; ETE, extrathyroid extension; HT, Hashimoto’s thyroiditis

**Fig. 5:**
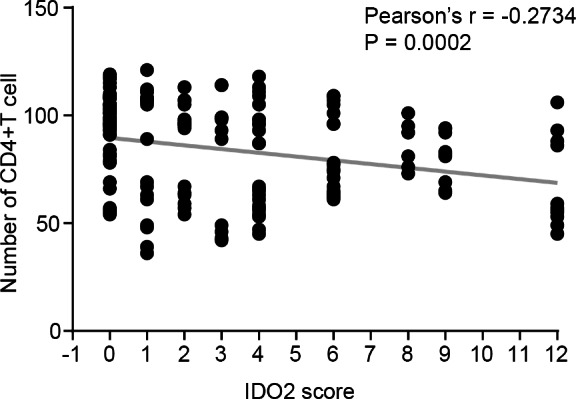
Association between the number of CD4 + T cells and the score of IDO2 expression

Although the infiltration of CD4 + T cells had no significant relationship with the clinicopathological features and prognosis of MTC patients, we found that IDO2 expression is negatively correlated with CD4 expression in MTC tissues. Correlation analysis revealed a reverse correlation between the above two parameters (Spearman’s r=-0.243, *P* = 0.001; Table 5). Additionally, the obviously opposite expression of the two indicators could be found in many MTC tissues (Fig. 4B C). A scatter plot describing the relationship between IDO2 score and the number of CD4 + T cells was shown in Fig. 5. Pearson’s correlation analysis also revealed a negative correlation between the two parameters (Pearson’s r = -0.2734, *P* = 0.0002). In conclusion, our results showed that IDO2 may have an inhibitory effect on the CD4 + T cell, similar to the previous studies.

## Disscussion

Although the prognosis of MTC patients with tumors confined to the thyroid is fairly good, no curative therapies are effective for patients with locally/locoregionally advanced inoperable disease and/or distant metastases [6]. Chemotherapy regimens can induce transient benefits in MTC, but their role as durable and/or curative therapies are limited [38]. The most effective regimen has been doxorubicin, alone or in combination with cisplatin, which achieved the response rate of approximately only 20% [39]. As for the molecular targeted therapies, recent treatments for MTC include multikinase inhibitors that target the RET and MAPK pathways. TKIs, such as vandetanib and cabozantinib, were approved in the United States for the treatment of symptomatic or progressive MTC in patients with unresectable locally advanced or metastatic disease. These two TKIs have achieved a significant increase in progression-free survival, and for patients with RET M918T-positive mutations, a considerable benefit of overall survival was observed [40–42]. Results of clinical trials revealed that MTC has a considerable degree of tumor heterogeneity, affecting its sensitivity to molecular targeted therapies. Moreover, although two new selective RET inhibitors, selpercatinib (LOXO-292) and pralsetinib (BLU-667), have brought durable clinical responses without notable off-target toxicities in RET-mutant MTCs [43, 44], more data on a larger number of patients in phase 2 and 3 prospective randomized clinical trials are needed. More importantly, the highly selective RET inhibitors will not be useful in patients who lack RET mutations (~ 45% of cases).

The role of immunotherapy in MTC is not fully studied [45]. MTC is not considered a very immunogenic tumor, which is a prerequisite for the efficacy of immunotherapy. Previous studies have identified evidence of T cell infiltration on MTC [46]. Recently, Nikita et al. [47] have reported that MTC is a more immunologically active tumor than previously reported. Findings that the presence of immune infiltrate is much more common in patients with MTC encourage further investigation of immune-based therapies for MTC. In recent years, anti-PD‐1/PD‐L1 therapy has shown promising clinical prospects in the treatment of thyroid carcinomas, including papillary thyroid carcinoma (PTC) and MTC. PD-L1 may be a potential biomarker for the management of patients with PTC [48, 49]. Moreover, Bi et al. reported that the PD-1/PD-L1 pathway was expressed in MTC patients and was significantly correlated with the distant metastases at the surgery [50]. Shi et al. demonstrate that PD‐L1 positivity was associated with aggressive clinicopathologic features and was independently predictive of structural recurrence and biochemical recurrence/persistent disease [51]. However, the role of several checkpoint inhibitors, including pembrolizumab and nivolumab (PD-1 inhibitors) as well as ipilimumab (CTlA-4 inhibitor) in the treatment of MTC are still unclear, resulting in the need for new biomarkers to amplify the effect of immune checkpoint inhibitors or to identify new and more efficient therapeutic targets.

IDO2 aroused the interest of many researchers since it was first discovered by Ball et al. in 2007 [26]. Given that IDO2 and IDO1 have a remarkable sequence homology, the primary efforts of the scientific community were addressed toward unveiling its role in the modulation of immune responses, assuming that a parallel analogy in the immunoregulatory functions was accompanied. Through the generation of IDO2 gene-deficient mice, many critical differences were detected between IDO2 and IDO1 [29]. However, a mutual influence came to light between the two paralogues regarding their expression and function. IDO2 was essential for IDO1-dependent induction of T regulatory cells. Meanwhile, a great many of the IDO2 transcripts were subjected to alternative splicing in IDO1 knockout mice [29]. Again, many studies further demonstrated the role of IDO2 in autoimmune arthritis [34, 52], Crohn’s disease [53], and psoriasis-like inflammation [54]. However, the results were complicated. One possible explanation is that the activity of IDO2 may be strictly related to the physiopathologic context and cellular microenvironment [55].

Subsequently, the researchers found that IDO2 may play a nonnegligible role in cancer progression by exerting many kinds of cancer-promoting effects [26, 30, 31, 56–58]. Mandarano et al. [30] found that high expression of IDO2 was significantly correlated with poor prognosis in patients with non-small-cell lung cancer. Moreover, a close relationship between IDO2 expression, increased PD-L1 levels, tumor-infiltrating lymphocytes localization, and NSCLC poor prognosis was detected, making it possible for IDO2 to be a potential prognostic biomarker or part of combined therapies with immune checkpoint inhibitors. Moreover, Yamasuge et al. [57] reported that IDO2 depletion could reduce tumor growth in a mouse model of Lewis lung carcinoma. IDO2 depletion altered the tumor microenvironment, leading to the enhancement of immune cell invasion, which suggests that IDO2 is an important immune regulator in the tumor microenvironment. Furthermore, the role of IDO2 in human pancreatic ductal adenocarcinoma (PDAC) has been extensively and deeply studied [31, 59]. The “IDO2-deficient status” significantly associates with improved disease-free survival in PDAC patients having received adjuvant radiotherapy, implying that IDO2 may represent an important drug target in PDAC therapy [31].

To the best of our knowledge, this is the first study about IDO2 immunohistochemical expression in MTC. In our study, using long-term follow-up data of this relatively rare disease from a single institution, we demonstrate clear evidence linking IDO2 to MTC degree of malignancy and potential immunomodulatory function. High expression of IDO2 is closely associated with more aggressive clinicopathological features, such as multifocality, ETE, a higher pT stage and especially a higher pN stage. At the same time, the expression level of IDO2 has nothing to do with the age range, heredity, unilateral and bilateral lesions, and maximum tumor size (Fig. 1B). The pT stage of MTC is associated with the maximum tumor size and extrathyroidal extension, but the results showed that IDO2 expression was not correlated with maximum tumor size. One possible explanation is that the difference is small concerning the degrees of malignancy when MTC is confined to the thyroid gland(T1/T2/T3a). In addition, the percentage of patients with maximum tumor size >4 was limited(5.3%), which may affect the accuracy of the analysis.

Of all the clinicopathological characteristics, the expression level of IDO2 had the most significant effect on lymph node metastasis. In the IDO2-low group, thirty-three (66.4%) patients presented as pN0 stage, whereas only fourteen (18.2%) patients in the IDO2-high group had no lymph node metastasis. In patients with lymph node metastases, the proportion of IDO2-high patients was much higher than its counterparts (63% vs. 37%). Given the significant correlation between IDO2 expression and the status of lymph node metastasis, we further examined the expression of IDO2 in the corresponding lymph nodes. IHC results indicated that the IDO2 expression of lymph node tissues was significantly related to the metastasis status (Table 5). Previous studies have not reported such a remarkable effect of IDO2 on lymph node metastatic status [60], suggesting that IDO2 may play distinct roles based on the pathophysiologic feature or immune microenvironment of different cancers. Consequently, these results indicated that IDO2 had great potential to play as a lymph node metastasis biomarker.

Moreover, survival analysis suggests that high expression of IDO2 tends to result in a worse prognosis in patients with MTC. Similar to Mandarano’s conclusion that high expression of IDO2 correlated with poor non-small-cell lung cancer prognosis [30], we found that there is a significant difference of RFS between IDO2-high and IDO2-low MTC patients ( Fig. 2B). Unfortunately, there was no significant difference in OS between the two groups (Fig. 2 A). One possible reason may be the insufficient follow-up period because the patients enrolled in our study all received surgery after 2010. The results require further follow-up.

Another surprising finding of IDO2 involvement is sexual dimorphism. Nevler et al. [31] have reported that the development of PDAC in IDO2 -/- female mice tends to be less than that in the male counterparts. In the meantime, female patients with PDAC rarely harbor the IDO2-deficient status, suggesting that IDO2 plays a more important carcinogenic role in female PDAC patients. However, in our study, the IDO2 expression level is almost equal in male MTC patients (49.4% versus 50.6%), whereas a relatively large gap of its expression exists in the female counterparts (66.7% versus 33.3%). Moreover, the pN stage was higher in male patients. Since female patients were more likely to be manifested as a IDO2-low status, this finding further validated the view that MTC patients with lymph node metastasis tend to have higher levels of IDO2 expression. Furthermore, IDO2 has a remarkable influence on the RFS of female patients (Fig. 2 F), whereas the OS and RFS of the male counterparts were hardly affected by IDO2 (Fig. 2 C and 2D). Together, these results all illustrated that female MTC patients may be more affected by IDO2 and thus should be taken into high consideration for immunotherapy involving IDO2 inhibition.

A number of studies have reported the effects of IDO2 on tumor immune resistance [33–37]. Similar to IDO1, IDO2 was also reported to inhibit the proliferation of CD4 + and CD8 + T cells [33, 34]. In our study, the CD4 + T cell infiltration was negatively correlated with IDO2 expression level (Table 5), which indirectly verified the above point of view. The level of CD4 + T cell infiltration has no impact on most of the clinicopathological characteristics, except for the heredity of MTC patients (Table 6). However, the low and high groups presented a striking difference in terms of IDO2 expression. Furthermore, the expression of CD4 had no significant effect on the OS/RFS of MTC patients (Fig. 4D and E). The above results give rise to a speculation that IDO2 may fulfill its immunomodulatory function partly by affecting the infiltration or proliferation of CD4 + T cells, which need to be further clarified.

The present study has several limitations. The main limitation of the work we present here is its retrospective population study design. Next, due to the relatively good prognosis of MTC, only 22 (11.8%) recurrences and 13 (6.9%) disease-related deaths have been observed; thus, the effect of IDO2 on prognosis may not be fully assessed. Moreover, the information of RET gene mutation was not available for all patients, so we couldn’t analyze the interaction between RET gene and IDO2 expression. Furthermore, we only observed the effect of IDO2 on the infiltration of CD4 + T cells, other effects on the immune environment of MTC caused by IDO2 remain to be seen. Finally, based on the previous experience of others, we determined the “high” and “low” expression groups of IDO2 and CD4 + T cell infiltration by ourselves, so errors can be made along the way.

In conclusion, we demonstrated that the expression level of IDO2 is associated with aggressive characteristics and is predictive of RFS and biochemical recurrence in patients with MTC. Moreover, although much less studied, IDO2 may represent an effective alternative drug target in cancer immunotherapy. The close relationship between IDO2 and CD4 + T cell infiltration in the MTC microenvironment, together with its potential prognostic implications, could open the way for the assessment of a possible therapeutic target with IDO2 selective inhibitors, both by discovering new pathogenic mechanisms and by exploring new pharmacological agents for MTC.

## Data Availability

The data that support the findings of this study are available on request from the corresponding author. The data are not publicly available due to privacy or ethical restrictions.
